# Head-to-head comparison of commercial artificial intelligence solutions for detection of large vessel occlusion at a comprehensive stroke center

**DOI:** 10.3389/fneur.2022.1026609

**Published:** 2022-10-10

**Authors:** Jacob Schlossman, Daniel Ro, Shirin Salehi, Daniel Chow, Wengui Yu, Peter D. Chang, Jennifer E. Soun

**Affiliations:** ^1^Center for Artificial Intelligence in Diagnostic Medicine, University of California, Irvine, Irvine, CA, United States; ^2^University of California Irvine School of Medicine, Irvine, CA, United States; ^3^Department of Neurology, University of California, Irvine, Irvine, CA, United States; ^4^Department of Radiological Sciences, University of California, Irvine, Irvine, CA, United States

**Keywords:** artificial intelligence, large vessel occlusion, machine learning, deep learning, stroke

## Abstract

**Purpose:**

Despite the availability of commercial artificial intelligence (AI) tools for large vessel occlusion (LVO) detection, there is paucity of data comparing traditional machine learning and deep learning solutions in a real-world setting. The purpose of this study is to compare and validate the performance of two AI-based tools (RAPID LVO and CINA LVO) for LVO detection.

**Materials and methods:**

This was a retrospective, single center study performed at a comprehensive stroke center from December 2020 to June 2021. CT angiography (*n* = 263) for suspected stroke were evaluated for LVO. RAPID LVO is a traditional machine learning model which primarily relies on vessel density threshold assessment, while CINA LVO is an end-to-end deep learning tool implemented with multiple neural networks for detection and localization tasks. Reasons for errors were also recorded.

**Results:**

There were 29 positive and 224 negative LVO cases by ground truth assessment. RAPID LVO demonstrated an accuracy of 0.86, sensitivity of 0.90, specificity of 0.86, positive predictive value of 0.45, and negative predictive value of 0.98, while CINA demonstrated an accuracy of 0.96, sensitivity of 0.76, specificity of 0.98, positive predictive value of 0.85, and negative predictive value of 0.97.

**Conclusion:**

Both tools successfully detected most anterior circulation occlusions. RAPID LVO had higher sensitivity while CINA LVO had higher accuracy and specificity. Interestingly, both tools were able to detect some, but not all M2 MCA occlusions. This is the first study to compare traditional and deep learning LVO tools in the clinical setting.

## Introduction

Acute ischemic stroke (AIS) remains a leading cause of disability and death worldwide (1). Large vessel occlusions (LVOs) in particular are associated with more severe presenting deficits and contribute disproportionately to higher rates of functional dependence and mortality (2). The impact of endovascular treatment for LVO is profound, with the number needed to treat to reduce disability for one patient as 2.6 (3). Expedited thrombectomy is critical, as every 15 min of improvement in door to revascularization times results in better rates of independent ambulation and functional outcomes (4). Thus, prompt diagnosis of an LVO is critical to select eligible patients and to allow for greater flexibility in patient transfer and treatment. The time dependence of acute stroke triage can be challenging for radiologists who may have busy worklists, but automated detection tools hold promise of screening and prioritizing positive LVO cases at the top of the worklist, allowing radiologists to diagnose the most time-sensitive patients first (5).

Commercial software for automated detection of LVO are increasingly being utilized in the clinical workspace. Some tools are based on traditional machine learning algorithms, while others use deep learning (6, 7). RAPID LVO (RAPID 4.9, iSchemaView, Menlo Park, CA) is a traditional machine learning model with demonstrated sensitivity and specificity of 97 and 74%, respectively (6). CINA LVO (Avicenna.ai, La Ciotat, France) is a deep learning model with demonstrated sensitivity and specificity of 98.1 and 98.2%, respectively (7).

Despite the availability of these commercial artificial intelligence tools for LVO detection, there is a lack of data comparing traditional machine learning and deep learning solutions in a real-world setting. To our knowledge, this is the first study to compare LVO tools in a comprehensive stroke center. The specific aim of this study is to compare and validate the performance of both RAPID LVO and CINA LVO for LVO detection in anterior circulation stroke and to characterize the limitations of each.

## Materials and methods

### Subjects

This was a retrospective, single center study performed at University of California, Irvine, a comprehensive stroke center, using anonymized data from December 2020 to June 2021. Inclusion criteria was as follows: (1) suspected acute stroke patients who had CT angiography (CTA) performed, (2) imaging done within 24 h of symptom onset, and (3) RAPID LVO output included with CTA acquisition. Patients who had either (1) imaging acquired at outside facilities or (2) technically inadequate CTA (e.g., poor contrast bolus, significant motion, or other artifact that would preclude evaluation by both human and automated assessment) were excluded. The study was HIPAA compliant and was approved by the local institutional review board (IRB). A waiver of written consent was granted by the IRB.

A total of 263 CTA cases (median age [IQR] = 68 years [IQR 56–79]; 122 females) for suspected stroke met the inclusion and exclusion criteria. Ten of these patients had isolated M2 middle cerebral artery (MCA) occlusions and were analyzed separately. A total of 253 CTA cases were included in the analysis.

### Imaging and automated LVO detection parameters

CTAs were acquired using three scanners from two vendors (Phillips & Siemens), including two 256-slice scanners with 128 detectors and a 128-slice scanner with 64 detectors. All CTA studies were performed as a single arteriovenous phase contrast study with a 60 mL intravenous contrast injection, injection rate of 5 mL/second using bolus tracking triggered from the aortic arch, slice thickness 1 mm, and coverage of the aortic arch to vertex.

RAPID LVO is an FDA approved tool for detection of intracranial internal carotid artery (ICA) and M1 MCA occlusions. The RAPID LVO algorithm is a traditional machine learning model which primarily relies on vessel density threshold assessment. After identifying the relevant anatomy with an anatomic template, the total sum of voxel densities for both large and small vessels are calculated and compared for anomaly detection (6).

CINA LVO is also an FDA approved LVO detection tool for ICA and M1 MCA occlusions. CINA LVO is an end-to-end deep learning tool implemented with multiple neural networks for detection and localization tasks.

### Study design

Ground truth was based off interpretation of the raw data from the CTA by radiology reports and confirmed by two neuroradiologists (9 and 10 years experience, respectively). For discrepancies, a third neuroradiologist provided adjudication (11 years experience).

**Figure 1:**
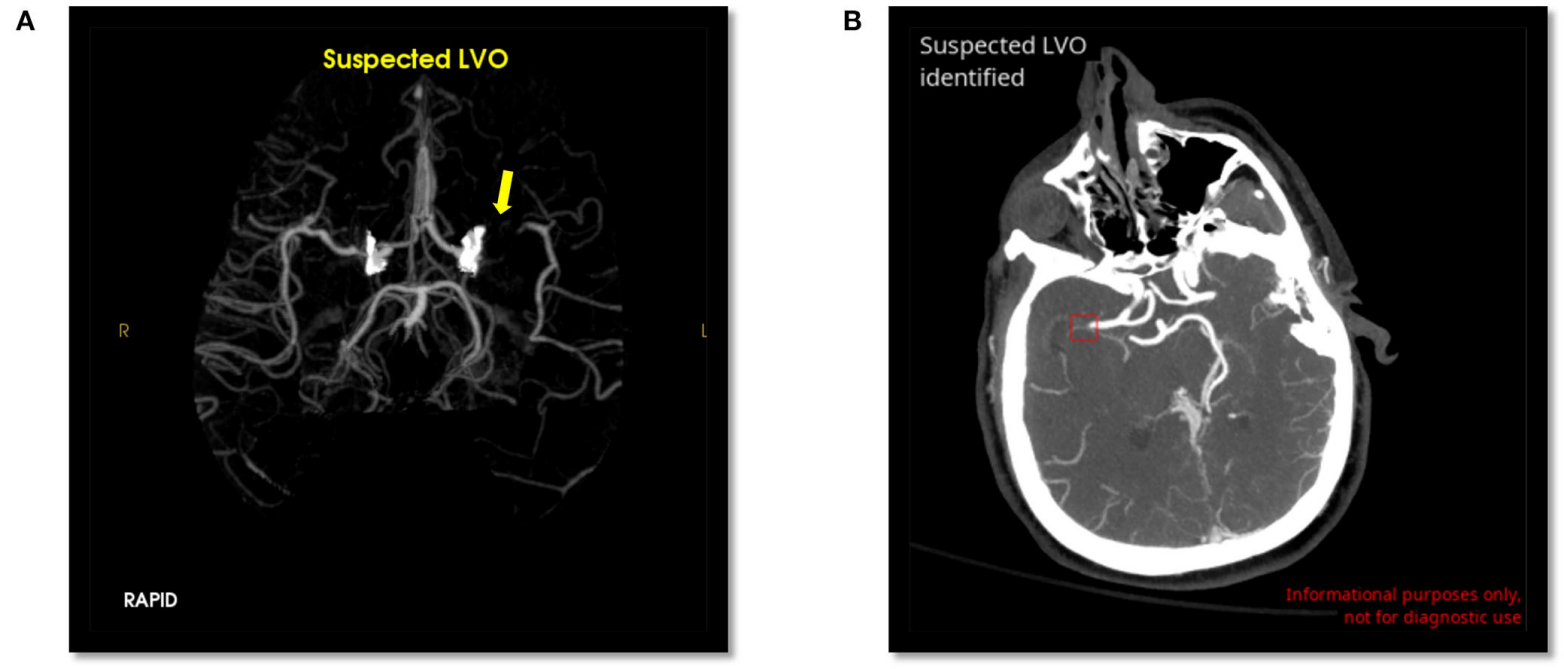
Positive LVO detected by RAPID LVO (**A**, yellow arrow, left M1 MCA) and CINA LVO (**B**, red box, right M1 MCA).

Scans were evaluated by RAPID LVO and CINA LVO, and the output and location of LVO (ICA, M1 MCA, or both) were recorded. Example positive LVO cases evaluated by both tools are shown in Figure 1. Performance metrics including accuracy, sensitivity, specificity, positive predictive value (PPV), and negative predictive value (NPV) were calculated from the output of both tools. For type 1 (false positive) and type 2 (false negative) errors, a visual inspection by the neuroradiologists was conducted to evaluate the potential reason for the error.

## Results

**Figure 2:**
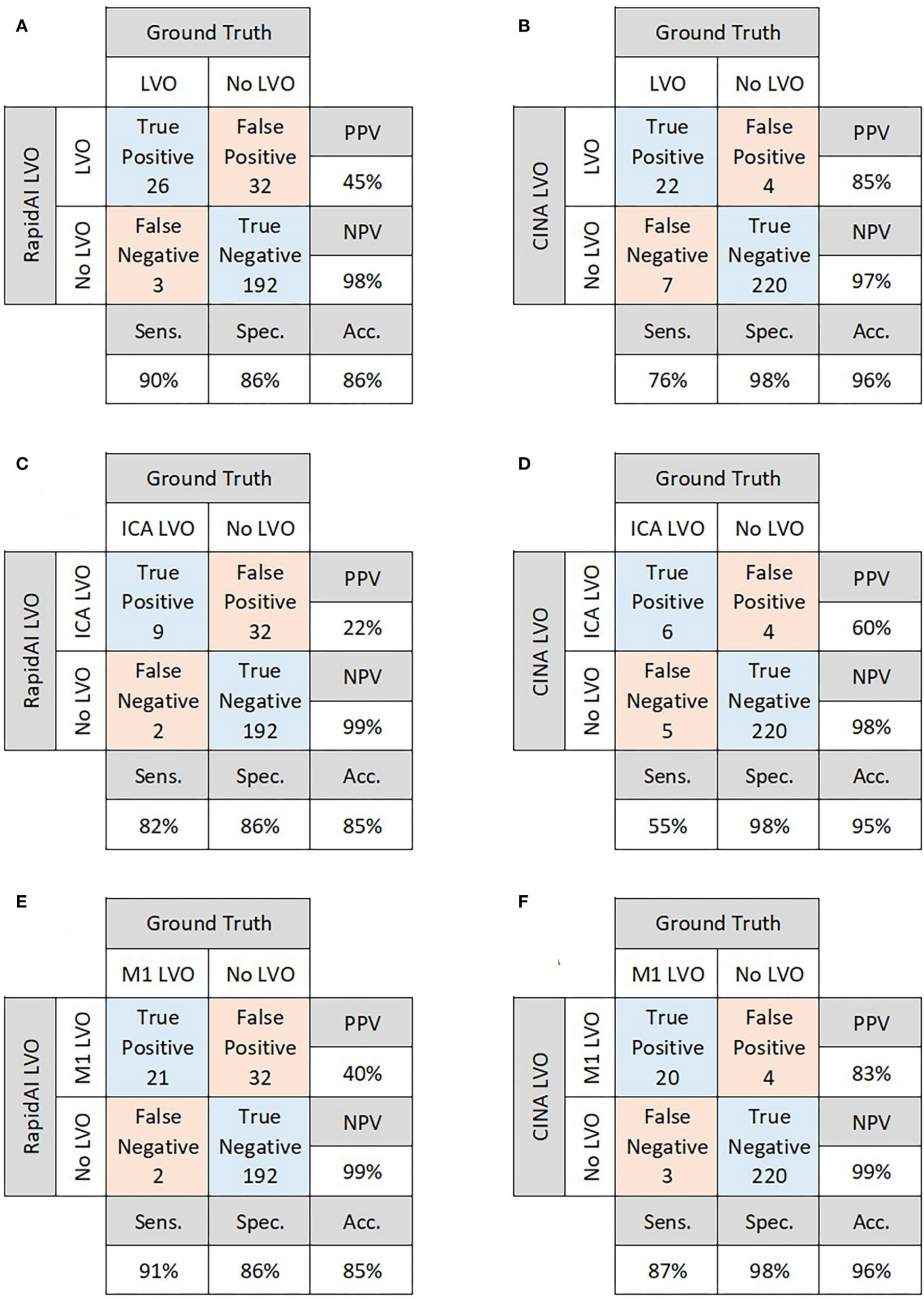
Confusion matrices for overall performance of RAPID LVO **(A)** and CINA LVO **(B)**, ICA occlusions detected by RAPID LVO **(C)** and CINA LVO **(D)**, and M1 MCA occlusions detected by RAPID LVO **(E)** and CINA LVO **(F)**.

There were 29 positive and 224 negative LVO cases. For overall performance, RAPID LVO demonstrated an accuracy of 0.86, sensitivity of 0.90, specificity of 0.86, PPV of 0.45, and NPV of 0.98, while CINA demonstrated an accuracy of 0.96, sensitivity of 0.76, specificity of 0.98, PPV of 0.85, and NPV of 0.97 (Figures 2A,B).

Out of the 29 positive LVO cases, there were 6 isolated ICA LVOs and 18 isolated M1 LVOs. There were 5 patients who were observed as having both an ICA and M1 LVO. For ICA LVOs, RAPID demonstrated a sensitivity of 0.82, while CINA demonstrated a sensitivity of 0.55. For M1 LVOs, RAPID demonstrated a sensitivity of 0.91, while CINA demonstrated a sensitivity of 0.87 (Figures 2C–F).

There were 10 M2 LVOs analyzed separately. RAPID successfully detected 8/10, while CINA successfully detected 3/10, for a demonstrated sensitivity of 0.8 and 0.3, respectively.

### Errors

**Figure 3:**
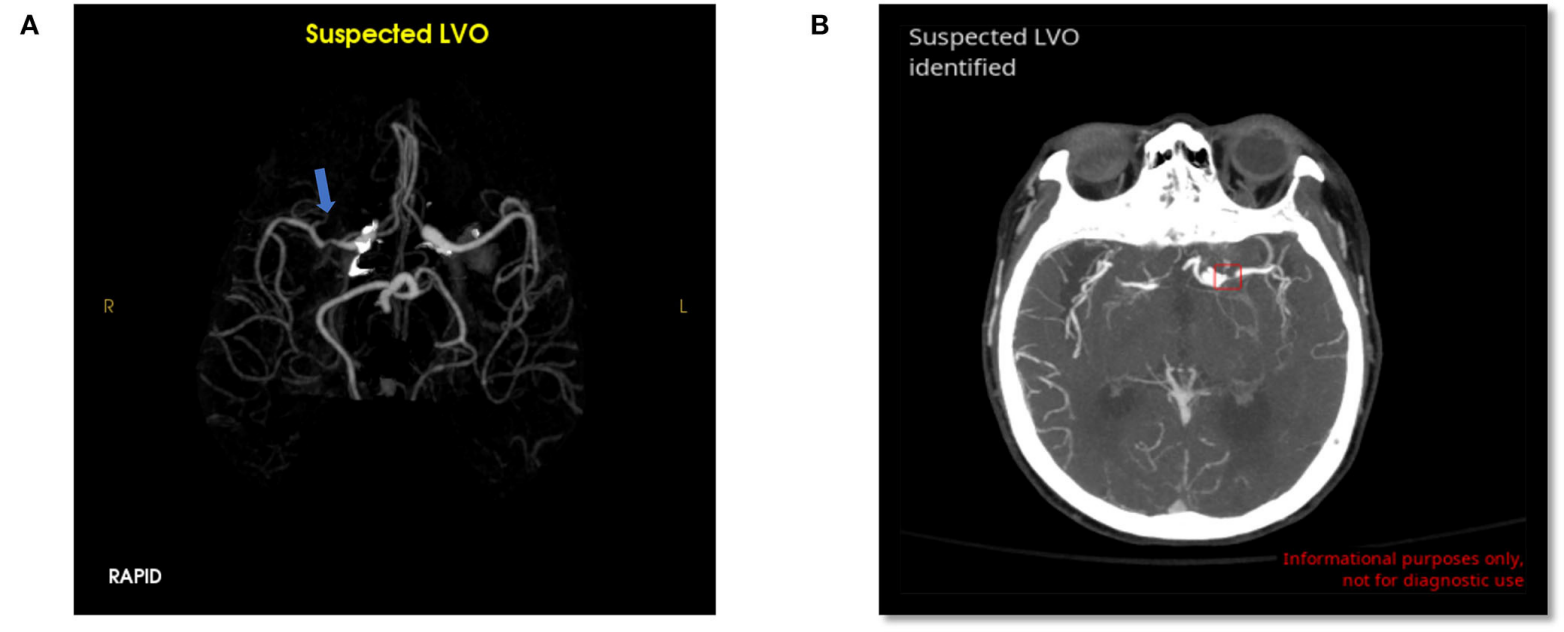
False positive LVO from RAPID LVO (**A**, blue arrow, right M1 MCA) and CINA LVO (**B**, red box, left M1 MCA). These type 1 errors were likely due to stenosis.

RAPID had 32 false positives (12.6%) and 3 false negatives (1.2%) for overall LVO detection. CINA had 4 false positives (1.6%) and 7 false negatives (2.7%). The possible reasons for errors are detailed in Table 1. Examples of false positives for both tools are shown in Figure 3.

**Table 1:** RAPID LVO and CINA LVO errors as evaluated by ground truth.

**False positive reason**	**RAPID LVO** **(n)**	**CINA LVO** **(n)**
Vessel caliber change (e.g., aneurysm, stenosis)	15	4
Contrast bolus timing	6	0
Mass effect	3	0
MCA asymmetry	2	0
Motion artifact	1	0
Bilateral occlusions, moyamoya	1	0
Postprocessing error (Bone)	3	0
Unknown reason	1	0
**False negative reason**	**RAPID LVO**	**CINA LVO**
ICA reconstitutes at terminus	1	1
Bilateral occlusions, moyamoya	2	1
Unknown reason	0	5

The most common false positive reasons for RAPID were vessel caliber change (47% of all non-LVO cases), contrast bolus timing (2.7%), and mass effect or postprocessing error (1.3% each). The most common false positive reasons for CINA were stenosis (0.9%) and caliber change (0.9%).

The most common false negative reasons for RAPID were cases with bilateral occlusions/moyamoya (6.9% of total true LVO cases) and the ICA reconstituting at the terminus of the patient (3.4%). The most common false negative reasons for CINA were unexplained misses (17.2% of true LVO cases), bilateral occlusions/moyamoya (3.4%), and the ICA reconstituting at the terminus (3.4%).

## Discussion

This is one of the first studies to compare the performance of traditional and deep learning LVO detection tools in the clinical setting. RAPID had overall higher sensitivity while CINA had overall higher accuracy and specificity. Both tools successfully detected the majority of ICA and M1 MCA occlusions. Interestingly, both tools were able to detect some, but not all M2 MCA occlusions even though neither is FDA approved for M2 LVO detection.

Prior RAPID LVO's validation reported a sensitivity and specificity of 97 and 74%, respectively, based on a cohort of 877 patients, 346 of which had a positive LVO (excluding M2 and M3/M4 occlusions) (6). By comparison, our study demonstrated RAPID LVO to have a sensitivity and specificity of 90 and 86%, respectively, based on a cohort of 253 patients. CINA LVO's validation reported a sensitivity and specificity of 98 and 98%, respectively, based on a cohort of 378 patients, 156 of which had a positive LVO (7); whereas, our study demonstrated CINA LVO to have a sensitivity and specificity of 76 and 98%, respectively.

The discrepancies for both tools could potentially be explained by the lower prevalence of LVOs in our real-world sample size, as compared to the samples used in both RAPID and CINA's validation papers (11% prevalence vs. 42 and 31%, respectively). Limitations of these tools in pathologies such as moyamoya and intracranial atherosclerotic disease (ICAD) may also explain discrepancies in performance at our institution.

Both tools mislabeled cases of moyamoya steno-occlusive disease with prominent leptomeningeal collaterals, contributing to both false positive and false negative cases. This is a potential limitation of these tools, particularly in areas that serve large Asian populations, as East Asian populations have a marked increase in incidence of moyamoya (8). Both tools also missed a case of ICA occlusion that reconstituted at the carotid terminus. Vessel caliber change from stenosis was the most common reason for false positives in both RAPID and CINA. This points to ICAD as a current limitation of both tools and could potentially result in higher rates of false positives when deployed at locations serving high-ICAD populations.

As both tools successfully detected most occlusions, their deployment could improve radiology workflow by shortening CTA report turnaround times and more importantly, door-to-treatment times through earlier notification of LVO results. These tools could make a difference at transfer centers and resource-limited sites, expediting transfer and triage for life-saving stroke therapies.

Future directions for these tools could include improving M2 and posterior circulation stroke detection, which would further improve patient triage. However, it is critical for these tools to balance sensitivity and specificity, as the dangers of increasing one at the cost of the other could lead to alert fatigue or missing patients with LVOs, respectively. There are ways for both tools to improve their detection and increase their PPV. A previous paper demonstrated RAPID's PPV could be improved by incorporating a relative vessel density threshold (9). No similar paper has demonstrated this for CINA LVO to the authors' knowledge and remains a potential opportunity for study.

One limitation of this study is that we did not evaluate the impact of these tools on clinical outcomes. Other studies have previously shown a significant improvement in transfer times of LVO patients (10), but there are mixed results at comprehensive stroke centers (9, 11–13). This study included a relatively small sample size at a single institution which may limit generalizability. It is also important to note we were unable to compare the code-level mechanisms behind the decision-making process for each tool, as the code is proprietary. As a result of this, we were unable to identify the reasons for some errors, and the errors were interpreted in the context of the patient's pathology. Furthermore, it is important to acknowledge that our ground truth, defined as two board-certified neuroradiologists (in addition to a third for adjudication), could have made the wrong judgement call due to human error.

Both RAPID and CINA LVO demonstrate high negative predictive values, as well as high sensitivity and specificity. This study compared both tools using real-world clinical data to highlight the differences in performance between traditional machine learning and deep learning-based programs. As more automated LVO detection tools become commercially available, it will be paramount to conduct comparison studies to evaluate their performance in the clinical setting.

## Data availability statement

The raw data supporting the conclusions of this article will be made available by the authors, without undue reservation.

## Ethics statement

The studies involving human participants were reviewed and approved by University of California, Irvine Institutional Review Board. Written informed consent for participation was not required for this study in accordance with the national legislation and the institutional requirements.

## Author contributions

JS: manuscript preparation, data gathering, and analysis. DC, WY, and DR: data gathering and analysis. SS: data analysis. PC: data analysis and software programming. JES: manuscript revision, study design, data gathering, and analysis. All authors contributed to the article and approved the submitted version.

## Funding

This study received funding from Canon Medical (JES). The funder was not involved in the study design, collection, analysis, interpretation of data, the writing of this article or the decision to submit it for publication.

## Conflict of interest

Author PC is co-founder of and owns stock in Avicenna.AI, has past and current research funding from and is a paid consultant for Canon Medical, has a current grant from Novocure, has past research funding from GE, and is a paid consultant and speaker for Siemens. Author DC had owns stock in Avicenna.ai and is a paid consultant for Canon Medical. Author JES had current research funding from the Radiological Society of North America Research Scholar Grant. The remaining authors declare that the research was conducted in the absence of any commercial or financial relationships that could be construed as a potential conflict of interest.

## Publisher's note

All claims expressed in this article are solely those of the authors and do not necessarily represent those of their affiliated organizations, or those of the publisher, the editors and the reviewers. Any product that may be evaluated in this article, or claim that may be made by its manufacturer, is not guaranteed or endorsed by the publisher.
